# Age at disability onset and self-reported health status

**DOI:** 10.1186/1471-2458-8-10

**Published:** 2008-01-09

**Authors:** Eric W Jamoom, Willi Horner-Johnson, Rie Suzuki, Elena M Andresen, Vincent A Campbell, the RRTC Expert Panel on Health Status Measurement

**Affiliations:** 1 College of Public Health and Health Professions, University of Florida, PO Box 100231 Gainesville, FL 32610, USA; 2 RRTC: Health & Wellness, Oregon Health & Science University, CDRC – PO Box 574, Portland, OR 97207, USA; 3 National Center on Birth Defects and Developmental Disabilities, Centers for Disease Control and Prevention, 1600 Clifton Rd.; MS-E-88; Atlanta GA 30333, USA

## Abstract

**Background:**

The critical importance of improving the well-being of people with disabilities is highlighted in many national health plans. Self-reported health status is reduced both with age and among people with disabilities. Because both factors are related to health status and the influence of the age at disability onset on health status is unclear, we examined the relationship between disability onset and health status.

**Methods:**

The U.S. 1998–2000 Behavioral Risk Factor Surveillance system (BRFSS) provided data on 11,905 adults with disability. Bivariate logistic regression analysis modeled the relationship between age at disability onset (based on self-report of duration of disability) and fair/poor self-perceived health status, adjusting for confounding variables.

**Results:**

Key variables included demographics and other measures related to disability and general health status. Disability onset after 21 years of age showed significant association with greater prevalence of fair/poor health compared to early disability onset, even adjusting for current age and other demographic covariates. Compared with younger onset, the adjusted odds ratios (OR) were ages 22–44: OR 1.52, ages 45–64: OR 1.67, and age ≥65: OR 1.53.

**Conclusion:**

This cross-sectional study provides population-level, generalizable evidence of increased fair or poor health in people with later onset disability compared to those with disability onset prior to the age of 21 years. This finding suggests that examining the general health of people with and those without disabilities might mask differences associated with onset, potentially relating to differences in experience and self-perception. Future research relating to global health status and disability should consider incorporating age at disability onset. In addition, research should examine possible differences in the relationship between age at onset and self-reported health within specific impairment groups.

## Background

"Disability is an issue that affects every individual, community, neighborhood, and family..."[1] People can acquire disabilities at any point in their lives. Of critical importance is the ability to develop or maintain a high quality of life after the acquisition of a disability [2]. Health related quality of life (HRQOL) is included as an overarching aspect of the American health strategic plan, *Healthy People 2010*, which is a set of national health objectives that encourages the use of self-rated health as a measure to evaluate health status in the population. Population-based surveillance of general health status monitors progress of two overall goals from *Healthy People 2010*: 1) to increase the quality and years of healthy life, and 2) to eliminate health disparities [3]. Surveillance questions on HRQOL can be used to examine different outcomes for people with and without disabilities and detect possible disparities [4]. However, even within specific impairments and diagnoses, people with disabilities report a broad range of self-reported health on commonly-used measures [5].

Factors that may impact self-reported health status include severity of disability or health condition, type of activity limitation, and age of the person with the disability [2,6]. Further, in what has been referred to as the "disability paradox", people with serious and persistent disabilities often report experiencing a good or excellent quality of life when to others it would appear that their health is poor [7]. This seeming paradox may be related to adoption of a positive disability minority group identity [8] or to a tendency on the part of outside observers to equate poor health with disability while people with disabilities may view them as separate constructs [9]. Age at onset of a disability, as well as the duration of the disability, can also impact health status [10]. Individuals who acquire a disability later in life may be more likely to rate their global health status in relation to their perceived health prior to the disability and have greater difficulty adjusting to the disability [11]. In contrast, early disability onset and longer duration of disability may allow greater adjustment to the disability both in terms of psychosocial identity development and adoption of coping strategies, leading to higher reported general health [12,13]. Evaluation of general health itself also may be adjusted to reflect changing standards and values in response to disability [12,14,15].

Empirical evidence supports the view that self-reported health status is related to age at onset and duration of disability. For example, people with congenital deafness have reported better health status than people with later onset deafness [16,17]. In people with spinal cord injury (SCI), both increasing age with SCI and more advanced age at injury onset have been associated with higher depression levels and poorer self-perceived health [18]. Other disease specific studies suggest earlier age onset is associated with better reported health status, even accounting for advancing age [10,19]. However, the overall relationship between age at onset and health status in broad population-based disability groups (e.g., among people with activity limitations or who use special equipment) has not been fully characterized. If the age that disability is acquired is associated with perceived general health, this knowledge might assist in more sensitive measurement of health, as well as in developing tailored interventions and interpreting heterogeneous age-related effects on general health status.

We analyzed data from the USA Behavioral Risk Factor Surveillance System (BRFSS) 1998–2000 to assess the relationship between disability onset and self-reported health status, a common measure of global health [20]. Specifically, we asked if general health status differs for people with different ages of disability onset, while controlling for possible confounders.

## Methods

The BRFSS is a state-based telephone (random-digit-dialed) survey of the noninstitutionalized U.S. population aged 18 years of age and older that provides data related to chronic diseases and their risk factors [21,22]. The BRFSS uses a Disproportionate Stratified Sample (DSS) method, where phone numbers are randomly selected throughout the state, business and nonworking numbers are omitted, and individuals aged 18 years and older are randomly selected from each household called. Data are subsequently weighted to reflect the complex sampling methods and nonresponse bias of the final sample [23]. This survey provides annual population-based cross-sectional data that can be used to analyze self-reported risks and health conditions. The BRFSS includes national "core" questions and modules, and state-added modules on special topics of interest to specific states. The BRFSS has previously been used to identify prevalence and correlates of general health among people with disabilities [5,24].

The present study analyzed data from the seven states and the District of Columbia that used both the core BRFSS Healthy Days measures (CDC HRQOL-4) and the HRQOL/Disability module each year from 1998–2000. The states (Arkansas, Iowa, Kansas, New York, North Carolina, Rhode Island, and South Carolina) and the District of Columbia represent a wide selection of the U.S. population. The total BRFSS sample size across all three years was 73,867. For the eight sites used in the study, response rates ranged from 52.2% (New York) to 75.1% (Kansas) with a median of 61.3% in 1998; in 1999, the range was 45.0% (New York) to 66.3% (Kansas) with a median of 48.7; in 2000, response rates ranged from 32.9% (New York) to 59.3% (North Carolina) with a median of 40.8% (see 2000 Behavioral Risk Factor Surveillance System Report [25] for response rates for each state in each year). For these analyses, we limited the sample to respondents who were classified as having a disability and answered a question on their disability duration (n = 11,905). Our working definition of disability was based on respondents saying "yes" to either of two questions: "Are you limited in any way in any activities because of any impairment of health problem?" or "Do you now have any health problem that requires you to use special equipment, such as a cane, a wheelchair, a special bed, or a special telephone?"

For this study, the dependent variable of self-reported general health status was classified as a dichotomous variable (fair/poor vs. excellent/very good/good), based on answers to the question "Would you say that in general your health is excellent, very good, good, fair, or poor?" This question is included in the CDC HRQOL-4, along with three questions about the number of recent days of the last 30 when physical health was not good, mental health was not good, and activities were limited because of poor health. The CDC HRQOL-4 questions have been demonstrated to predict morbidity, health care use, and mortality and are associated with chronic diseases and disability [26-28]. The retest reliability of HRQOL questions is moderate to excellent [4]. The questions also have demonstrated reliability and validity for population health surveillance [4,28,29] and people with disability [30].

The primary predictor variable for these analyses was age at disability onset, computed from questions on self-reported disability duration and current age. Respondents who answered yes to one or more of the disability screener questions above were asked how long their activities had been limited. Responses were given in days, weeks, months, or years. These responses were recoded by the authors to indicate the number of years, or portions of a year (in decimal format), activities had been limited. This disability duration variable was subtracted from the respondent's current age on the date of the interview to determine the respondent's age at the time of disability onset. Age at disability onset was then categorized into four groups: birth through 21 years of age, ages 22 to 44, ages 45–64, and age 65 years or older. We classified early disability onset as birth through age 21 years based on federal laws designating developmental disability services for individuals aged 0–21 years. Adult onset was considered age 22 years and older; the additional divisions within this age range were made to allow examination of potential effects of early adult versus older adult disability onset. Thus, there were four groups classified by differing ages at disability onset, based on information reported at time of interview.

In addition, we included a set of potential confounding variables: age (as a continuous variable); gender; race/ethnicity (white non-Hispanic, African American non-Hispanic, other non-Hispanic groups, and all Hispanics); current employment status (employed, unemployed, student/homemaker, retired, unable to work); education (< high school graduate vs. ≥ high school graduate); marital status (married, separated/divorced, widowed, never married); and disability duration (years limited as a continuous variable).

Descriptive analyses compared characteristics of all four disability onset groups and respondents who were classified as not having a disability. Logistic regression models with health status as the outcome report odds ratios (OR) and 95% confidence intervals (95% CI) for people with disabilities only. Models were constructed by forcing in age at onset as the primary predictor variable, and including additional variables if they had a meaningful effect on the odds ratio of age at onset (10% or more change in OR) or if the variable itself was a significant predictor of general health status. We performed an exploratory analysis to investigate whether the relationship of age at onset and health status might be different for men versus women by adding an interaction term between gender and age at onset, but the interaction was non-significant. An interaction of age and disability duration was also tested; there was no significant interaction (data available on request). Thus, results are provided for main effects only. Descriptive results were analyzed using SUDAAN 9.0.0 (Research Triangle Institute, Research Triangle Park, NC, 2004) for weighted data, and logistic regression was conducted with SPSS Complex Samples 14.0 for Windows (SPSS, Inc., Chicago, IL, 2005). Stratification and weighting variables related to the BRFSS sampling and weighting strategy were included in the analyses as design variables. This study was approved by the Institutional Review Boards at the University of Florida and Oregon Health & Science University.

## Results

There were 11,905 respondents who were classified as having a disability, which yielded a population estimate of 4,370,174 adults with disability for the seven states and the District of Columbia. These included people with computed disability onset between birth and age 21 years (raw n = 1,272), ages 22 and 44 (n = 4,085), ages 45 and 64 (n = 3,906), and age 65 years or older (n = 2,642). Table 1 compares characteristics of these groups and 58,483 adults who were not classified as having a disability. In general, these figures demonstrate a trend of increasing fair or poor reported health status across the age at onset groups. The early onset group was younger on average and more likely to be male, employed, and more educated compared to the later onset groups.

**Table 1:** Sample characteristics by age at onset

	Age at disability Onset Groups											
	
	0–21		22–44		45–64		≥65		No Disability		Total	
Sample Size	n = 1,272		n = 4,085		n = 3,906		n = 2,642		n = 58,483		n = 70,388	

Estimated Population	557,969		1,550,869		1,401,986		859,351		23,673,140		28,043,314	

**Variables**	Percent	(SE)	Percent	(SE)	Percent	(SE)	Percent	(SE)	Percent	(SE)	Percent	(SE)

Self-reported general health status:												
Fair or poor health (Age Adjusted)	24.59	2.16	40.72	1.48	52.64	1.46	55.97	1.88	9.02	0.23	14.18	0.24
Mean disability limitation years (sd)	23.28	0.94	9.21	0.29	6.93	0.18	3.75	0.12				
Mean current age (sd)	35.22	0.77	43.26	0.30	60.87	0.25	77.14	0.24	43.72	0.13	45.42	0.12
Current age ≥ 65	5.46	1.01	4.34	0.48	32.43	1.39	100.00	0.00	15.31	0.26	17.98	0.26
Gender: female	48.77	2.53	52.57	1.47	58.28	1.45	66.13	1.75	51.61	0.38	52.38	0.35
Race:												
White, non-Hispanic	73.53	2.44	75.06	1.44	78.59	1.30	85.49	1.53	74.44	0.36	75.00	0.33
Black, non-Hispanic	14.09	1.83	12.55	0.98	12.27	0.92	9.54	1.23	13.73	0.26	13.47	0.24
All Hispanic	8.23	1.77	8.98	1.19	6.55	0.93	4.03	0.95	7.95	0.27	7.82	0.24
Other	4.15	1.12	3.41	0.59	2.59	0.58	0.94	0.46	3.88	0.18	3.71	0.16
Income (n = 58,784): <$25000	42.01	2.63	40.80	1.46	49.59	1.59	61.37	2.19	26.87	0.37	30.05	0.34
Education: HS graduate or above	83.44	1.85	85.91	0.97	76.14	1.23	71.50	1.63	88.83	0.26	87.40	0.24
Marital status:												
Married or unmarried couple	46.53	2.51	59.62	1.40	62.97	1.34	44.69	1.90	60.39	0.37	59.72	0.34
Separated or divorced	12.71	1.59	19.31	1.03	16.15	0.93	7.66	0.97	10.95	0.22	11.61	0.20
Widowed	3.78	0.91	4.41	0.54	14.79	0.93	43.93	1.84	6.00	0.15	7.47	0.16
Never been married	36.98	2.50	16.65	1.16	6.09	0.67	3.71	0.69	22.65	0.34	21.20	0.30
Employment status (n = 70,306):												
Employed or self-employed	62.55	2.41	52.80	1.44	27.78	1.32	4.74	0.96	69.69	0.35	64.53	0.33
Unemployed	7.56	1.48	9.45	0.89	4.42	0.57	0.42	0.20	3.51	0.14	3.87	0.14
Retired	5.98	1.06	6.71	0.67	39.55	1.44	88.75	1.23	15.58	0.26	18.34	0.26
Student or homemaker	12.49	1.73	7.93	0.72	5.64	0.65	3.59	0.55	10.21	0.24	9.70	0.22
Unable to work	11.42	1.42	23.10	1.28	22.60	1.20	2.50	0.61	1.01	0.09	3.57	0.13
Top five major impairments (n = 11,169)† :												
Back or neck problem (n = 2109)	14.78	1.86	27.80	1.36	16.98	1.21	8.19	0.99				
Eye/Vision problem (n = 321)	7.43	1.48										
Arthritis/rheumatism (n = 1766)	5.53	1.09	10.35	0.85	19.92	1.21	26.27	1.81				
Fractures, bone or joint (n = 1033)	12.23	2.01	11.03	1.02	8.52	0.87	8.46	1.05				
Lung problem (n = 913)	13.32	1.72	6.32	0.67	9.58	0.98						
Walking problem (n = 838)			4.64	0.62			11.90	1.34				
Heart problem (n = 873)					11.10	0.97	11.02	1.25				

† The top five impairments or conditions selected on 1998–2000 BRFSS Disability/HRQOL module; Respondents were allowed to select from 14 major impairments or conditions, including ''other'' (n = 2232) for impairments or conditions not listed.

BRFSS = Behavioral Risk Factor Surveillance System, sd = standard deviation, HS = high school, SE = standard error

Table 2 provides the final adjusted parsimonious model of disability onset and fair/poor health. When controlling for current age, disability duration, gender, race/ethnicity, education, marital status, and employment, each of the adult onset groups was significantly more likely to report fair/poor health compared to the early onset group. The following variables also showed a significant relationship with fair/poor health: age; African American race/ethnicity; less than high school education; divorced/separated marital status; and not currently being employed. No significant association between disability duration and self-reported fair or poor general health was observed after adjusting for current age.

**Table 2:** Model of Predictors of fair/poor general health from the 1998–2000 BRFSS*

Variables		Adjusted OR	95% CI
Age at Onset:	0–21	reference	---
	22–44	1.52	(1.37, 1.68)
	45–64	1.67	(1.44, 1.94)
	≥65	1.53	(1.26, 1.86)
Age:	(per year increase)	1.02	(1.02, 1.02)
Duration:	(per year increase)	1.00	(0.99, 1.00)
Gender:	Male	reference	---
	Female	1.00	(0.95, 1.05)
Race/ethnicity:	White, non Hispanic	reference	---
	Black, non Hispanic	1.22	(1.15, 1.30)
	All Hispanic	1.01	(0.90, 1.14)
	Other	0.75	(0.69, 0.82)
Education:	≥ High school graduate	reference	---
	< High school graduate	1.84	(1.74, 1.95)
Marital Status:	Married/unmarried couple	reference	---
	Divorced/separated	1.39	(1.32, 1.47)
	Widowed	1.02	(0.96, 1.09)
	Never Been Married	0.99	(0.92, 1.07)
Employment Status:	Employed	reference	---
	Unemployed	2.57	(2.33, 2.83)
	Student/homemaker	1.90	(1.75, 2.06)
	Retired	1.92	(1.77, 2.09)
	Unable to Work	6.76	(6.29, 7.27)

*Logistic regression of BRFSS data are limited to 7 states & the District of Columbia and weighted with SPSS Complex Samples 14.0 (unweighted n = 11,734; estimated weighted n = 12,895,725).

OR = Odds Ratio CI = Confidence Interval; BRFSS = Behavioral Risk Factor Surveillance System

## Discussion

This study represents the first major stride to consider the relationship between different ages of disability onset and self-reported general health of a broadly defined, sizeable population of people with disabilities. While the BRFSS is a cross-sectional survey, this study contains information about a past "exposure" (disability onset) calculated from information reported at the time of the interview. The potential for recall bias regarding disability duration and ultimately our measure of age at disability onset is an inherent limitation of the data available. Given the cross-sectional nature of the survey data and analyses, as well as the reconstruction of prior disability onset, the causal inference of our findings is limited.

In our descriptive analysis, early age at onset (age < 22 years) was associated with better health status. Our regression results also characterize individuals with an early onset disability as reporting better general health than people with later onset disability, even when controlling for confounding variables, especially current age. These results may support those of previous studies with more homogeneous samples of specific impairments [10,16-19] and are consistent with theoretical models on adaptation to disability [12,13]; however, an alternative explanation may be that health conditions that occur more commonly in later life (e.g., arthritis, diabetes) may be associated with both disability and decreased health status. There are qualitative differences in many conditions that give rise to disability in early life compared with those that result in disability in later life. For instance, congenital conditions such as cerebral palsy may result in communication and ambulation limitations but not necessarily poor self-defined and self-reported health. Based on these findings, subsequent research should consider the timing of disability in addition to the presence of disability. More global examination of health status of people with disabilities may mask differences associated with age at disability onset.

Future directions include investigation of early versus later disability onset within specific conditions in a population-based sample, as well as comparisons of social support and life satisfaction among disability onset groups. In addition, the reasons for the association between age at onset and self-perceived health status should be investigated directly. Response shift is one theoretical reason for the difference [15], however, there are no direct measures of response shift in the BRFSS. Future research may need to examine this possible explanation, for example using Rapkin and Schwartz's "appraisal" measure [31]. In an ad-hoc analysis, we included life satisfaction as an indirect proxy of differences in self-appraisal in our model and found no appreciable changes (data available on request). As with other possible mechanisms that will require more detailed measurements, additional explanatory information (e.g., social networks, disability identity) should be used to further investigate age at onset differences.

These findings are subject to various limitations. The BRFSS sampling frame excludes institutionalized adults, restricting the inclusion of some individuals with severe disability who may report poorer general health. The phone survey methodology excludes people who are deaf or hard of hearing and potentially some individuals with severe mobility disabilities that limit their ability to answer the phone [32]. In addition, participation in the BRFSS, even among those sampled, has continued to decline in tandem with the secular trends of research in general [33]. Respondents from only seven states and the District of Columbia were included in the analyses. While the sample included respondents from the Eastern, Southern, and Midwestern regions of the country, these data may not be fully representative of adults with disabilities in the entire U.S. Furthermore, "disability" status and disability duration were determined by self-report and may be prone to subjective interpretation of respondents. The regression model did not control for limitation or impairment type due to the multiple constructs contained within the type of impairment question (impairments, diagnoses/diseases, activity limitations, and injuries) and the lack of response categories that were applicable to all respondents; specifically, the difference in response patterns between younger and older age at onset groups (see table 1) leave a large response group of heterogeneous conditions ("other") that differed across onset groups. Lastly, as noted previously, the study was cross-sectional. While we included age at onset as the prior historical exposure, retrospective construction of a "cohort" of this kind based on respondent recall data has inherent limitations to cause and effect interpretations (i.e., the pseudo cohort directly acts as a proxy based on the nature of this sample being cross-sectional). This strategy allowed initial examination of the relationship of age at onset and self-reported health status for individuals with a wide range of current ages and durations of disability, and provides support for future longitudinal studies to study these issues prospectively.

Despite listed weaknesses, the BRFSS has many strengths as a data source, including the population-based sampling methodology. Disability was defined broadly, increasing the generalizability of the results. The broad definition may have attenuated the effect size, however, since the age at onset relationship may be limited to subsets of people with disabilities. The substantial sample size of the dataset provided the ability to determine a moderate effect of age at onset with statistical precision.

## Conclusion

In this study, 23.3% of respondents with early onset disability reported having fair or poor health, while higher proportions of respondents with later disability onset reported fair/poor health. Despite adjusting for known confounders (e.g., current age, education), age at onset was significantly associated with reduced health status. These findings suggest age at disability onset may impact self-reported general health and should be considered when analyzing HRQOL differences within people with disabilities.

## Competing interests

The author(s) declare that they have no competing interests.

## Authors' contributions

EWJ contributed to the design of the study and had primary responsibility for data analysis and manuscript preparation. WHJ and VAC contributed to the study design, data analysis, and manuscript preparation. RS and EMA, contributed to the study design and participated in manuscript preparation. This study grew out of work from the REP, which provided valuable contributions toward the preparation of this article. All authors have read and approved the final manuscript.

## Pre-publication history

The pre-publication history for this paper can be accessed here:


